# Targeting BRD4 mitigates hepatocellular lipotoxicity by suppressing the NLRP3 inflammasome activation and GSDMD-mediated hepatocyte pyroptosis

**DOI:** 10.1007/s00018-024-05328-7

**Published:** 2024-07-09

**Authors:** Fangyuan Chen, Shuyu Li, Min Liu, Cheng Qian, Zhiyin Shang, Xu Song, Wei Jiang, Chuantao Tu

**Affiliations:** 1 Department of Gastroenterology and Hepatology, Zhongshan Hospital, Fudan University, Shanghai, 200032 China; 2 Department of Gastroenterology, Shanghai Public Health Clinical Center, Fudan University, Shanghai, 201508 China; 3 Institute of Neuroscience and State Key Laboratory of Neuroscience, CAS Center for Excellence in Brain Science and Intelligence Technology, Chinese Academy of Sciences, Shanghai, 200031 China; 4 Department of Pathology, Shanghai Public Health Clinical Center, Fudan University, Shanghai, 201508 China; 5 Department of Gastroenterology and Hepatology, Zhongshan Hospital (Xiamen), Fudan University, Shanghai, 361015 China

**Keywords:** Cell death, Liver inflammation, Histone acetylation, Mitochondria, IL-1β

## Abstract

Nod-like receptor family pyrin-containing protein 3 (NLRP3) inflammasome plays a pathologic role in metabolic dysfunction-associated steatohepatitis (MASH), but the molecular mechanism regulating the NLRP3 inflammasome activation in hepatocellular lipotoxicity remains largely unknown. Bromodomain-containing protein 4 (BRD4) has emerged as a key epigenetic reader of acetylated lysine residues in enhancer regions that control the transcription of key genes. The aim of this study is to investigate if and how BRD4 regulated the NLRP3 inflammasome activation and pyroptosis in MASH. Using the AML12 and primary mouse hepatocytes stimulated by palmitic acid (PA) as an in vitro model of hepatocellular lipotoxicity, we found that targeting BRD4 by genetic knockdown or a selective BRD4 inhibitor MS417 protected against hepatosteatosis; and this protective effect was attributed to inhibiting the activation of NLRP3 inflammasome and reducing the expression of Caspase-1, gasdermin D (GSDMD), interleukin (IL)-1β and IL-6. Moreover, BRD4 inhibition limited the voltage-dependent anion channel-1 (VDAC1) expression and oligomerization in PA-treated AML12 hepatocytes, thereby suppressing the NLRP3 inflammasome activation. Additionally, the expression of BRD4 enhanced in MASH livers of humans. Mechanistically, BRD4 was upregulated during hepatocellular lipotoxicity that in turn modulated the active epigenetic mark H3K27ac at the promoter regions of the *Vdac* and *Gsdmd* genes, thereby enhancing the expression of VDAC and GSDMD. Altogether, our data provide novel insights into epigenetic mechanisms underlying BRD4 activating the NLRP3 inflammasome and promoting GSDMD-mediated pyroptosis in hepatocellular lipotoxicity. Thus, BRD4 might serve as a novel therapeutic target for the treatment of MASH.

**Graphic abstract:**

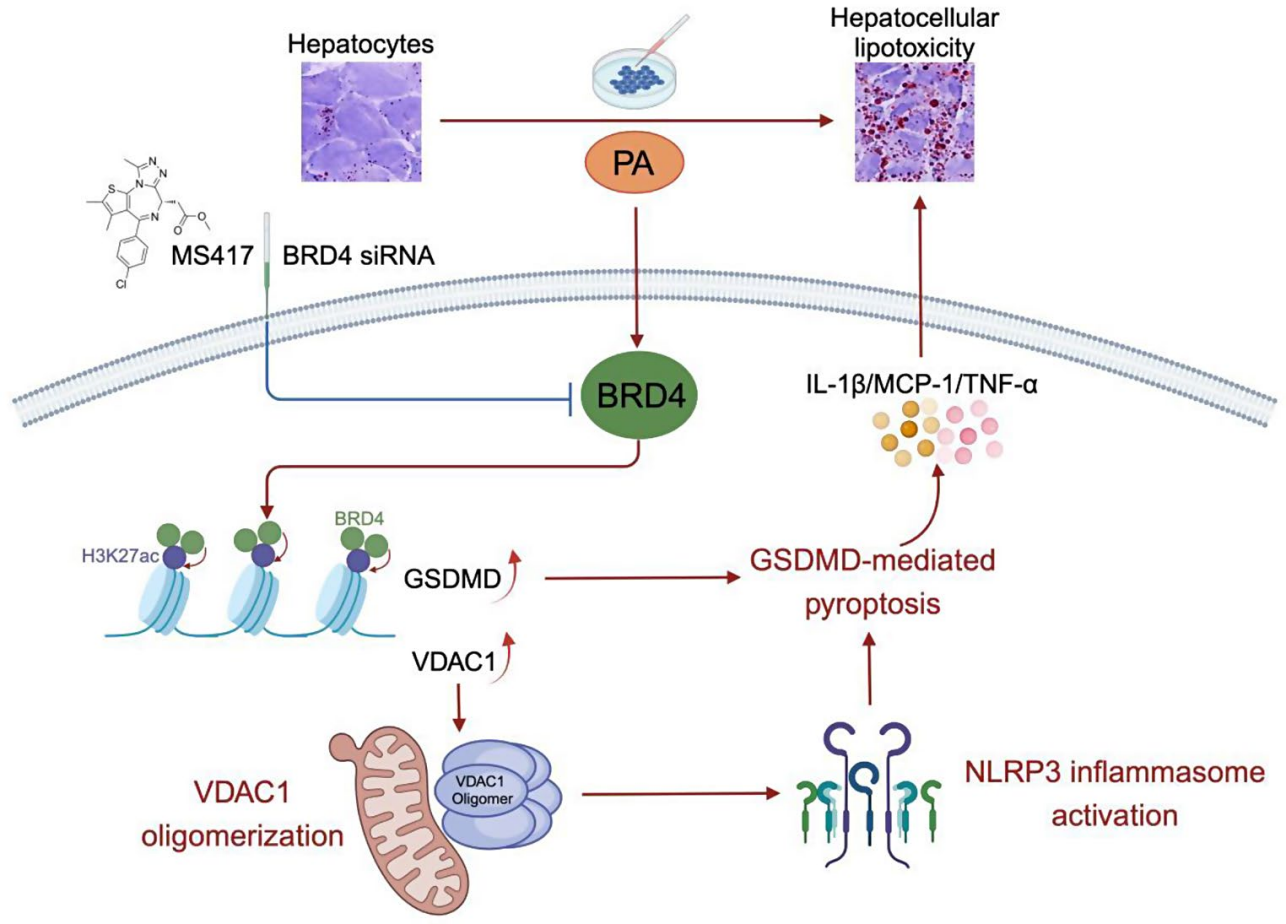

**Supplementary Information:**

The online version contains supplementary material available at 10.1007/s00018-024-05328-7.

## Introduction

Metabolic dysfunction-associated steatotic liver disease (MASLD), previously termed nonalcoholic fatty liver disease (NAFLD), has become the most prevalent chronic liver disease worldwide, affecting 20-25% of the global population [1, 2]. If left untreated, MASLD can progress to a more severe stage of metabolic dysfunction-associated steatohepatitis (MASH), previously known as nonalcoholic steatohepatitis (NASH), which is characterized by necroinflammation and different degrees of fibrosis [1–3]. Particularly, hepatic fibrosis is the histologic hallmark of MASH that can gradually progress to cirrhosis, liver failure, and eventually hepatocellular carcinoma (HCC) [3]. Despite MASLD posing a global health threat, there are still limited agents to treat or reverse MASH in clinical practice due to poorly understood the mechanism underlying the pathological alteration in MASH. Therefore, a greater understanding of the pathogenesis in MASLD and MASH is critical to identify novel strategies for combating this metabolic disorder.

Hepatosteatosis is the first hit during MASLD development and progression that can result from an imbalance between lipid acquisition and lipid disposal, leading to hepatocellular lipotoxicity and death [1, 4]. Hepatocytes damage and death initiate and facilitate liver inflammatory response that represent a central mechanism involved in the pathogenesis of MASLD [1–3]. Importantly, hepatocellular lipotoxicity is intimately associated with chronic liver inflammation, which is a characteristic of conditions predisposing to MASLD and MASH [5]. However, the mechanistic details of hepatocellular lipotoxicity during MASLD progression have not been fully elucidated.

Emerging evidences have shown that the NLRP3 inflammasome-mediated inflammatory cytokines play a key role in the pathogenesis of metabolic disorders such as diabetes, obesity, and MASLD [6–9]. The NLRP3 inflammasome has been shown to sense metabolites such as palmitic acid (PA), uric acid, and cholesterol crystals and its activation trigger the release of the proinflammatory cytokines interleukin (IL)-1β and IL-18 [6, 7]. Notably, saturated fatty acids have been shown to activate the NLRP3 inflammasome and sensitize hepatocytes to endotoxin response, promoting the pathological progression of MASH [6, 10]. NLRP3 inflammasome could induce inflammatory caspases, thereby driving classical or non-classical pyroptosis [9–12]. It has been well known that pyroptosis is a type of lytic programmed cell death characterized by the plasma membrane via gasdermin protein family members, leading to the subsequent release of inflammatory signals [9–13]. Of note, cleaved-GSDMD serves as a key executor of pyroptotic cell death that plays a pathogenic role in MASLD development and progression in both humans and mice, and targeting GSDMD markedly alleviated steatohepatitis in mouse models of MASH [9–11]. Thus, inhibiting the NLRP3 inflammasome activation and hepatocyte pyroptosis represent potential therapeutic strategies for mitigating MASH. Notably, it has been reported that the outer mitochondrial membrane protein VDAC1 is essential for the activation of NLRP3 inflammasome that regulates both cell metabolism and apoptosis at the mitochondrial level [14, 15]. Moreover, VDAC1 oligomerization is required for recruitment of NLRP3 into close association with VDAC and then promotes inflammasome activation [15]. However, mechanisms underlying the regulation VDAC1/NLRP3 inflammasome signaling axis during in MASH pathology are still largely understood.

Recent studies have shown that epigenetic modifications, including histone acetylation, implicate in the pathogenesis of MASLD and MASH genesis [2, 16]. Bromodomain-containing protein 4 (BRD4) is a member of the bromodomain and extraterminal domain (BET) family that functions as an epigenetic reader protein that has been known to bind to acetylated histones and nonhistone proteins and activate gene transcription by stabilizing binding of coactivators, mediators, and RNA polymerase II [17, 18]. Accumulating evidences have shown that BRD4 contributes to several diseases by modulating pyroptotic cell death, such as acute gouty arthritis, cerebral ischaemia, and colon injury [19–21]. BRD4 was also reported to implicate hepatic lipid metabolism, fibrosis, and HCC [22–24]. However, the role and epigenetic mechanism of BRD4 in MASLD remain insufficiently understood. Therefore, we aimed to investigate the effects of BRD4 on hepatocellular lipotoxicity, as well as to explore whether BRD4 is involved in activating the NLRP3 inflammasome and promoting hepatocyte pyroptosis during MASLD pathogenesis.

## Materials and methods

### Agents and antibodies

Reagents in this study were obtained as following. MS417 (#HY-111,139) from MedChemExpress (New Jersey, USA); DMEM/F12 (#ZQ600) from Zhong Qiao Xin Zhou Biotechnology (Shanghai, China); Oil Red O (ORO) staining kit (#0843) from ScienCell (California, USA); BODIPY 493/503 (#D2191), EGS (#21,565), GeneRuler 50 bp (#SM0373), 16% Formaldehyde Solution (#28,906), ChIP-Grade Protein A/G Magnetic Beads (#26,162) and RNase A (#EN0531) from Thermo Fisher Scientific (Boston, USA); Palmitic acid (#P9767) from Sigma-Aldrich (Missouri, USA); Albumin BovineV (fatty acid-free; #A8850) and Nigericin sodium salt (#IN1880) from Solarbio (Beijing, China); 4’,6-diamidino-2-phenylindole (DAPI; #C1006), Protein inhibitor cocktail (#P1005), Lipo8000 transfection reagent (#C0533), DAB Horseradish Peroxidase Color Development Kit (#P0203), RIPA lysis buffer (#P0013B), and Enhanced BCA protein assay kit (#P0010) from Beyotime (Shanghai, China); RNAiso Plus from Takara (#108-95-2, Tokyo, Japan); GoScript™ Reverse Transcription Mix and Random Primers (#A2800) and Eastep™ qPCR Master Mix (#LS2068) from Promega (Wisconsin, USA); Viability dye 506 (#62210-00) from Biogems (California, USA); 0.5 M EDTA (#7011), and 10X Glycine (#7005S) from Cell Signaling Technology (Boston, USA); 6X Gel loading dye (#B7024S), Micrococcal Nuclease (#M0247S), NEBNext Ultra™ II DNA Library Prep Kit for Illumina (#E7645S) and Multiplex Oligos for Illumina (#E7600s) from New England Biolabs (Ipswich, USA); AMPure XP (#A63881) from Beckman Coulter (California, USA). Penicillin, Streptomycin, and trypsin-ethylenediaminetetraacetic acid from Genom Biotechnology (Shanghai, China). All antibodies in this study were listed in Table S1.

### Isolation and culture of primary hepatocytes

From wild-type C57BL6/J mice, primary hepatocytes were isolated using the collagenase perfusion approach that has been previously described [8]. In summary, newly extracted hepatocytes were seeded into collagen I-coated petri plates and were cultivated in RPMI medium 1640 supplemented with 1.0 g/L glucose, 10% fetal bovine serum (FBS), 100 units/mL penicillin, and 100 mg/mL streptomycin at 37 ℃ in a humidified environment. Six hours after plating, the medium was changed, and the cells received the appropriate treatment.

### Cell treatment and transfection

Mouse hepatocyte cell line AML12 obtained from Zhong Qiao Xin Zhou Biotechnology (Shanghai, China) were cultivated in DMEM/F12 containing 10% FBS, 50 U/mL penicillin, and 50 µg/mL streptomycin. The cells were incubated in a humidified environment composed of 95% air and 5% CO_2_ at 37 ℃. AML12 or primary hepatocytes were seeded in 6-well plates in serum-containing media and were cultured to 70-80% confluence followed by serum-starved for 24 h. Subsequently, AML12 or primary hepatocytes were induced by 500 µM PA as an in vitro model of hepatocellular lipotoxicity [25–27]. Briefly, PA was firstly conjugated to bovine serum albumin (BSA) [27]; then was added in the medium to induce oxidative stress and lipoapoptosis in AML12 or primary hepatocytes.

MS417, a selective inhibitor of the BET-specific protein BRD4, was chosen to apply in AML12 or primary hepatocytes. Therefore, after the cells reached 70-80% confluence and were starved for 24 h, AML12 or primary hepatocytes were challenged with 500 µM PA or BSA (control) in serum-containing media for 24 h, with or without MS417 (50 nM).

AML12 or primary hepatocytes were transfected with siRNA for BRD4 to transiently knock down BRD4. Non-targeted scrambled control siRNA (NC siRNA) and 3 groups of BRD4 siRNA (#1, #2, and #3) were transfected into AML12 cells using Lipo8000 transfection reagent according to the manufacturer’s instruction. The BRD4 siRNA and NC siRNA were provided by Genepharma (Shanghai, China), and the produced oligo sequences are displayed in Table S2. BRD4 knockdown efficiency was verified by qPCR and Western blotting, respectively (Fig. S1). With the highest knockdown efficiency, siBRD4 (#1) was finally chosen to transfect AML12 hepatocytes. 48 h after transfection, cells were starved and then incubated with BSA (control) or PA accompanied by MS417 or not for another 24 h.

### Human liver specimens

Human liver specimens were obtained from patients with MASLD/MASH who underwent liver biopsy or surgical liver resection, and healthy liver tissues were obtained from the unaffected part of the liver from patients undergoing surgery for hepatic hemangioma or trauma repair. All liver samples came from Shanghai Public Health Clinical Center. Subjects with other chronic liver diseases were excluded, including viral hepatitis, cholestatic liver disease, excessive alcohol consumption, Wilson disease, hemochromatosis, drug-induced liver disease, and α-1-antitrypsin deficiency. All subjects gave written informed consent according to the Declaration of Helsinki, and the protocol, approved by ethical committees from Shanghai Public Health Clinical Center (#2022-S104-01).

### MASH liver samples

Mice liver samples including MASH and the control group were obtained from our recent cohort study [28]. In brief, male C57BL/6J mice aged eight weeks (Shanghai Slack Laboratory Animal Co., Ltd., Shanghai, China) were maintained in an SPF facility with an environmental control system. Mice had unrestricted access to food and liquids with a 12-hour light-dark cycle. Mice were fed either a normal chow diet (NCD) or a choline-deficient, L-amino acid-defined, high-fat diet (CDAHFD; Research Diets, USA) for 8 weeks [28].

### Histopathology and immunohistochemistry

For histopathological assessment, paraffin-embedded formalin-fixed liver sections were stained with hematoxylin and eosin (H&E), Masson’s trichrome, or Sirius red according to standard protocols [28]. Liver histology was blinded reviewed by two independent expert pathologists and MASH was defined by the NAFLD activity score (NAS) as follow [27]. Briefly, steatosis (0–3), lobular inflammation (0–3), and hepatocellular ballooning (0–2); and NAFLD activity score (NAS) was expressed as the sum of each scoring. Additionally, liver fibrosis and lipid droplet areas were also assessed blindly using ImageJ software based on Sirius red and H&E-stained sections, respectively.

Immunohistochemical (IHC) staining was carried out as our previously described [28, 29]. Briefly, 5 μm-thick sections were de-paraffinized, rehydrated, and subjected to heat-induced epitope retrieval. Then, slides were blocked and incubated overnight at 4 ℃ in the following primary antibodies (dilution 1:100): anti-BRD4, anti-VADC1, anti-NLRP3, and anti-GSDMD. The list of the antibodies is provided in Table S1. Following washing in PBS, sections were subsequently incubated with HRP-conjugated secondary antibodies (dilution 1:100) and developed using DAB Horseradish Peroxidase Color Development Kit according to the manufacturer’s protocol. Finally, the positive areas were examined under a light microscope (BX43, Olympus). Immunostaining signaling was quantified at a predetermined threshold using free NIH ImageJ.

### Immunofluorescence

The hepatocytes were dehydrated at 37 ℃ for 30 min and fixed with 4% paraformaldehyde for 30 min followed by permeabilization using 0.2% TritonX-100 in PBS. Then nonspecific binding was blocked with 5% BSA for 1 h at RT, followed by incubation with primary antibodies for BRD4 (dilution 1:100) and HNF4 (dilution 1:100) at 4 ℃ overnight. After washing in PBS, the cells were incubated with secondary conjugated antibodies for 1 h at 37 ℃. DAPI was used for nuclear staining. The slides were finally imaged by a confocal microscope (FV3000, Olympus).

### Oil red O and BODIPY staining

Lipid accumulation was analyzed using an Oil Red O (ORO) staining kit as described in the manufacturer’s protocol. AML12 cells or primary hepatocytes were washed with PBS, and fixed with 4% paraformaldehyde for 15 min. Then, cells were stained with freshly diluted 0.6% ORO solution preheated to 65 ℃ for 1–5 min. Cell nuclei were stained by hematoxylin for 10 s. The stained lipid droplets were observed under a light microscope (BX43, Olympus).

For BODIPY staining, the cells were treated and fixed the same with ORO staining. Then the fixed cells were stained with BODIPY 493/503 dye (dilution 1:1000) at RT for 10 min and protected from light, and the nuclei were stained with DAPI. The stained lipid droplets were observed under a confocal microscope (FV3000, Olympus). The areas of the lipid droplets in different groups were analyzed using NIH Image J 1.49 software.

### Western blot assay

Western blot analysis was performed according to standard protocols as previously described [28, 29]. Cells and liver tissues lysed in RIPA buffer were centrifuged at 4 ℃, and protein concentrations were assessed using the Enhanced BCA protein assay kit. Protein (10–20 µg) was resolved by 12.5% SDS-PAGE gel electrophoresis and transferred onto polyvinylidene fluoride membranes. The membranes were blocked and incubated overnight at 4 ℃ with primary antibodies (dilution 1:1000) against BRD4, β-Actin, NLRP3, total and cleaved GSDMD, pro- and cleaved-Caspase-1, IL-1β, apoptosis-associated speck-like protein (ASC), VDAC1, and H3K27ac. Followed by washing in TBST, the membranes were incubated with a 1:5000 dilution of peroxidase-conjugated secondary antibodies under RT for 1 h. β-actin was used as loading control. Bands were visualized with ECL™ Western Blotting Detection Reagents (EpiZyme, Shanghai, China), and the optical density of the bands was determined using the NIH ImageJ software.

### VDAC cross-linking assay

Briefly, AML12 hepatocytes treated as indicated were washed twice with PBS, and incubated with Ethylene Glycol-bis (Succinimidylsuccinate, EGS), a chemical cross-linking reagent wich diluted in PBS, at 30 ℃. Then the lysed protein samples from AML12 cells were subjected to SDS-PAGE and immunoblotting analyzed with anti-VDAC1 antibody. After time gradient and concentration gradient experiments (Fig. S2A, B), we finally selected 600 µM EGS for 30 min as the optimal incubation conditions [30].

### RNA isolation and real-time quantitative PCR

Total RNA was extracted using RNAiso Plus according to the manufacturer’s instructions [28]. For qPCR, total RNA was reverse transcribed to cDNA using Promega GoScript™ Reverse Transcription Mix and Random Primers. Relative quantitative gene expression levels were measured by quantitative PCR using Eastep™ qPCR Master Mix on QuantStudio 5 System (Applied Biosystems). β-actin was used as an internal control, and the relative expression of target genes was calculated using the 2^−ΔΔCT^ method. All primers used are listed in Table S3.

### Flow cytometry analysis

Cell viability was measured using fixable Viability dye 506. Briefly, AML12 cells grown in six-well plates were treated as indicated and harvested. Then, cells were resuspended in PBS at a concentration of 1–10 × 10^6^/mL. Add 1 µL of Viability Dye per 1 mL of cells, then vortex immediately, after that cells were incubated for 15 min at RT in the dark. Cells were washed three times with PBS buffer before testing, and flow cytometry was employed to detect cell viability. The Viability Dye 506 can be excited by the violet (405 nm) laser and has a peak emission of 506 nm detectable by the 510/50 bandpass filter. The dead cells were identified by positive signals. Data were analyzed using the FlowJo software (V10.8.0).

### Chromatin immunoprecipitation (ChIP)

For ChIP, AML12 cells were seeded in 15 cm culture dishes and were challenged with PA with or without the co-culturation of MS417. For ChIP, protease inhibitor cocktail (#P1005, Beyotime) was used in all buffers. Cells were PBS washed and fixed for 10 min at RT with shaking in fresh methanol-free 1% formaldehyde, then quenched by 0.125 M glycine. Cells were lysed in lysis buffer LB1 (50mM Hepes-KOH, 140mM NaCl, 1mM EDTA, 10% Glycerol, 0.5% NP-40, 0.25% Triton X-100) and LB2 (10mM Tris-HCl, 200mM NaCl, 1mM EDTA, 0.5mM EGTA) and incubated at 4 ℃ for 10 min, respectively. Then cells were resuspended in LB3 (10mM Tris-HCl, 200mM NaCl, 1mM EDTA, 0.5mM EGTA, 0.1% Na-Deoxycholate, 0.2% SDS) and were sonicated to shear DNA to 250–500 bp. The sonicated samples were centrifuged at 10,000 rcf, 10 min at 4 ℃, then the supernatant was taken and the DNA concentration was detected. The samples containing 5–10 µg of DNA were taken and incubated with anti-BRD4, anti-H3K27ac antibody (1 µl per 5 µg samples), or BSA for input DNA overnight at 4 ℃.

The next day, ChIP-Grade Protein A/G Magnetic Beads (20 µl per 5 µg samples) were added to each ChIP sample at RT and incubated at 4 ℃ for 2 h with rotation to collect chromatin/antibody. Beads were collected with a magnet and washed in RIPA buffer (1% Triton X-100, 0.1% Na-deoxycholate, 0.1% SDS, TE), RIPA buffer with 5 M NaCl, LiCl buffer (0.25 M LiCl, 0.5% NP-40, 0.1% Na-deoxycholate, TE), TE with 0.2% Triton X-100 and TE, succeedingly. After proteinase K digestion and reverse crosslinking, the precipitated DNA and input DNA were purified using a PCR purification kit and subjected to DNA library construction.

For ChIP-sequence (ChIP-seq) library preparation, samples were performed using NEBNext Ultra™ II DNA Library Prep Kit and NEBNext Multiplex Oligos for Illumina according to the manufacturer’s recommendations. DNA libraries were sequenced on an NOVAseq600 instrument, with 150-bp paired-end sequencing [31].

### ChIP-seq analysis

Raw reads were filtered using cutadapt (version 1.15). Clean reads were aligned to the mouse reference genome GRCm38 (mm10) using Bowtie2 aligner (version 2.3.3.1) with default parameters. MACS2 was used to call the broad peaks with -q 0.05 [31]. Differentially bound peaks were identified with DiffBind (version 3.13) [32]. The peaks were annotated with the annotatedPeaks.pl command from HOMER (version 4.9) to associate peaks to their nearest genes. BigWig files were generated using the bamCoverage command from deeptools (version 3.5.0) with -smoothLength 50 –binSize 5 and –scaleFactor, and markdup.bam files were as input for bamCoverage [32]. The scaleFactors were normalization factors calculated from DiffBind based on the number of all mapped reads that fall into the called peak regions. ChIP-seq heatmaps and profile plots were generated with computeMatrix followed by the plotHeatmap command from deeptools (version 3.5.0). Merged BigWig files of PA/Vehicle-anti H3K27ac, PA/MS417-anti H3K27ac, PA/Vehicle-input, and PA/MS417-input were generated from two biological replicates with the bigWigMerge command followed by the bedGraphToBigWig command from UCSC [32]. The gene set enrichment analysis for differentially bound genes was conducted using KEGG pathway analysis.

### Statistical analysis

All data are presented as the mean ± standard deviation (SD). Statistical analyses were performed using GraphPad Prism 9.0 (La Jolla, CA, USA). Statistically significant differences between two independent groups were made by unpaired two-tailed Student’s *t*-test. Comparisons among multiple groups were performed by one-way ANOVA with Tukey’s *post hoc* honest significant difference test or two-way ANOVA followed by Tukey’s or Sidak’s multiple comparisons test. Clinical characteristics and NAS scores were corrected using Spearman’s rank correlation test. For all comparisons, significance is indicated as *P < 0.05, **P < 0.01, ***P < 0.001, and ****P < 0.0001; ns indicates not significant.

## Results

### Targeting BRD4 limits the accumulation of lipid droplets in hepatocellular lipotoxicity

**Fig. 1:**
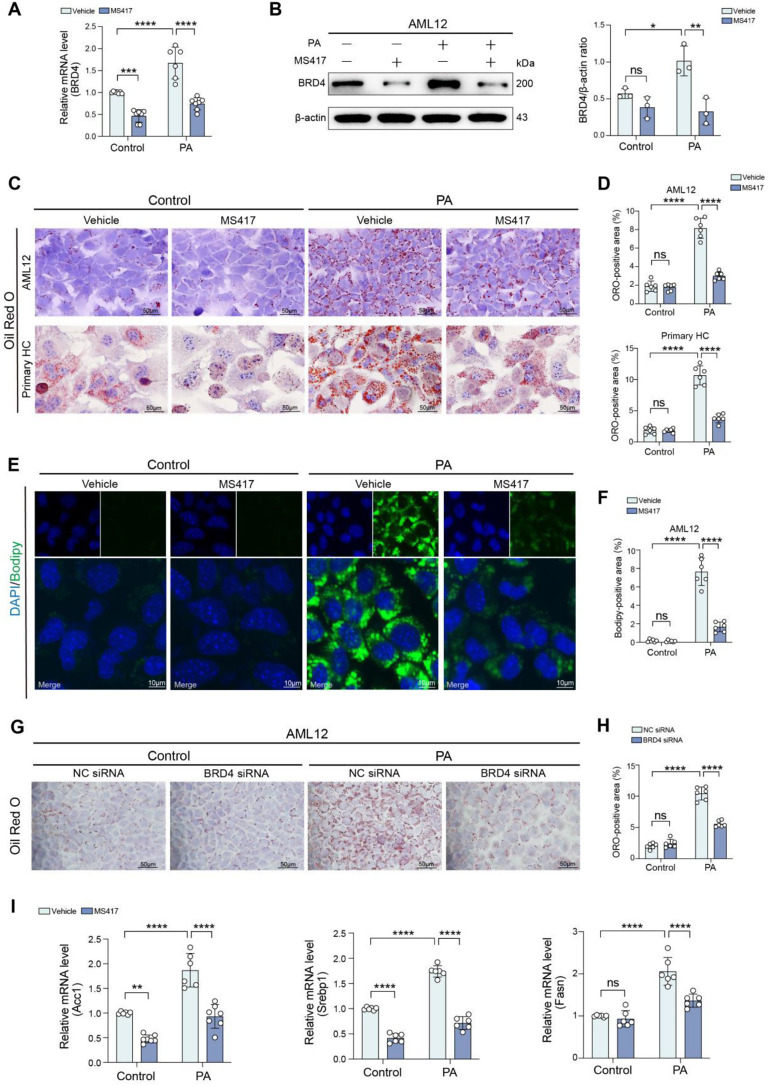
Targeting BRD4 limits the accumulation of lipid droplets in hepatocellular lipotoxicity. **A, B** AML12 hepatocytes were exposed to palmitic acid (PA, 500µM) or BSA (control) for 24 h in the presence MS417 (50nM) or vehicle. **A** Quantitative RT-PCR was performed to determine the mRNA levels of BRD4 in the hepatocytes. Results were normalized to β-actin mRNA. **B** BRD4 protein in cell lysate was determined using Western blot (left); β-actin was used as loading control and results normalized to β-actin (right). **C** Representative microphotographs of ORO-staining of AML12 (upper line) or mouse primary hepatocytes (HC) (bottom line). The cells were exposed to PA or BSA for 24 h in the presence MS417 or vehicle. Scale bar: 50 μm. **D** Lipid droplets accumulation in AML12 or primary hepatocytes was quantified based on **(C)**. **E** Representative microphotographs of BODIPY staining (green) in AML12 hepatocytes, which were exposed to PA for 24 h in the presence of MS417 or vehicle. Nuclei were stained with DAPI (4’,6-diamidino-2-phenylindole, blue). Scale bar: 10 μm. **F** Intracellular lipid droplets was quantified based on **(E)** (*n* = 6). **G** Representative microphotographs of ORO-staining of AML12 hepatocytes from indicated treatment. AML12 hepatocytes were transfected with non-targeted scrambled control siRNA (NC siRNA) or siRNA targeting BRD4 (BRD4 siRNA). 48 h after transfection, cells were exposed to PA or vehicle for 24 h. Scale bar: 50 μm. **H** Lipid droplets accumulation in cells was quantified based on **G** (*n* = 6). **I** AML12 hepatocytes were exposed to PA or BSA (control) for 24 h in the presence MS417 or vehicle. Quantitative RT-PCR was performed to determine the mRNA expression of *Acc1*, *Srebp1*, and *Fasn*. Results were normalized to β-actin mRNA and expressed as fold change compared to control group. Statistical significance is denoted by *p < 0.05, **p < 0.01, ***p < 0.001; ****p < 0.0001; ns indicates not significant

To assess the effects of BRD4 on lipid accumulation in hepatocytes during MASLD progression, AML12 or primary mouse hepatocytes were exposed to 500 µM PA to induce an in vitro model of hepatocellular lipotoxicity [25–27]. Our results showed that after 24 h of exposure, PA remarkably induced the expression of BRD4 in AML12 hepatocytes as compared with BSA-treated cells. However, these increased levels of BRD4 mRNA and protein expression were effectively blocked by the treatment of the selective BRD4 inhibitor MS417 (50 nM) (Fig. 1A, B). We next examined whether BRD4 contributes to the development of hepatosteatosis and lipotoxicity; the accumulation of lipid droplets in hepatocytes was documented morphologically using ORO staining in PA-induced hepatocytes. As expected, PA overload led to a greatly increased deposition of lipid droplets in AML12 hepatocytes as well as primary hepatocytes, but MS417 administration remarkably abrogated this PA-induced hepatocellular lipotoxicity (Fig. 1C). In line with these observations, MS417 treatment reduced the area of lipid droplets in both AML12 and primary hepatocytes stimulated by PA (Fig. 1D). Similarly, BODIPY staining also confirmed the protective effect of MS417 on the deposition of lipid droplets in PA-treated AML12 hepatocytes (Fig. 1E, F).

We further analyzed the effect of modifying the abundance of BRD4 on hepatocellular lipotoxicity in cultured AML12 hepatocytes. AML12 hepatocytes were transfected with a siRNA-mediated knockdown of BRD4 or with an NC siRNA. After 48 h, the transfected cells were challenged with overload PA for another 24 h. As expected, knockdown BRD4 in AML12 hepatocytes effectively downregulated the expression of BRD4 at both the mRNA and protein levels (Fig. S1A). PA-treated AML12 hepatocytes led to increasing the deposition of lipid droplets as confirmed by staining of ORO and its quantification; however, this increased lipid droplets in AML12 hepatocytes challenged by PA were abrogated by siRNA-mediated knockdown of BRD4 (Fig. 1G, H).

To investigate the intracellular mechanism of how BRD4 blockage affects the lipid accumulation in PA-induced hepatocyte lipotoxicity, we assessed the mRNA levels of the key enzymes involved in liver lipogenesis and mitochondrial β-oxidation, including acetyl-CoA carboxylase 1 (*Acc1*), sterol regulating element-binding protein-1 (*Srebp1*), and fatty acid synthase (*Fasn*). Our results showed that the levels of *Acc1*, *Srebp1*, and *Fasn* mRNA were increased in PA-induced AML12 hepatocytes as compared with that in BSA-treated AML12 hepatocytes; however, these upregulated genes were significantly suppressed by administration with MS417 (Fig. 1I).

Altogether, our results indicate that targeting BRD4 by genetic knockdown or MS417 protects against hepatocellular lipotoxicity.

### Targeting BRD4 suppresses the activation of NLRP3 inflammasome during hepatocellular lipotoxicity

**Fig. 2:**
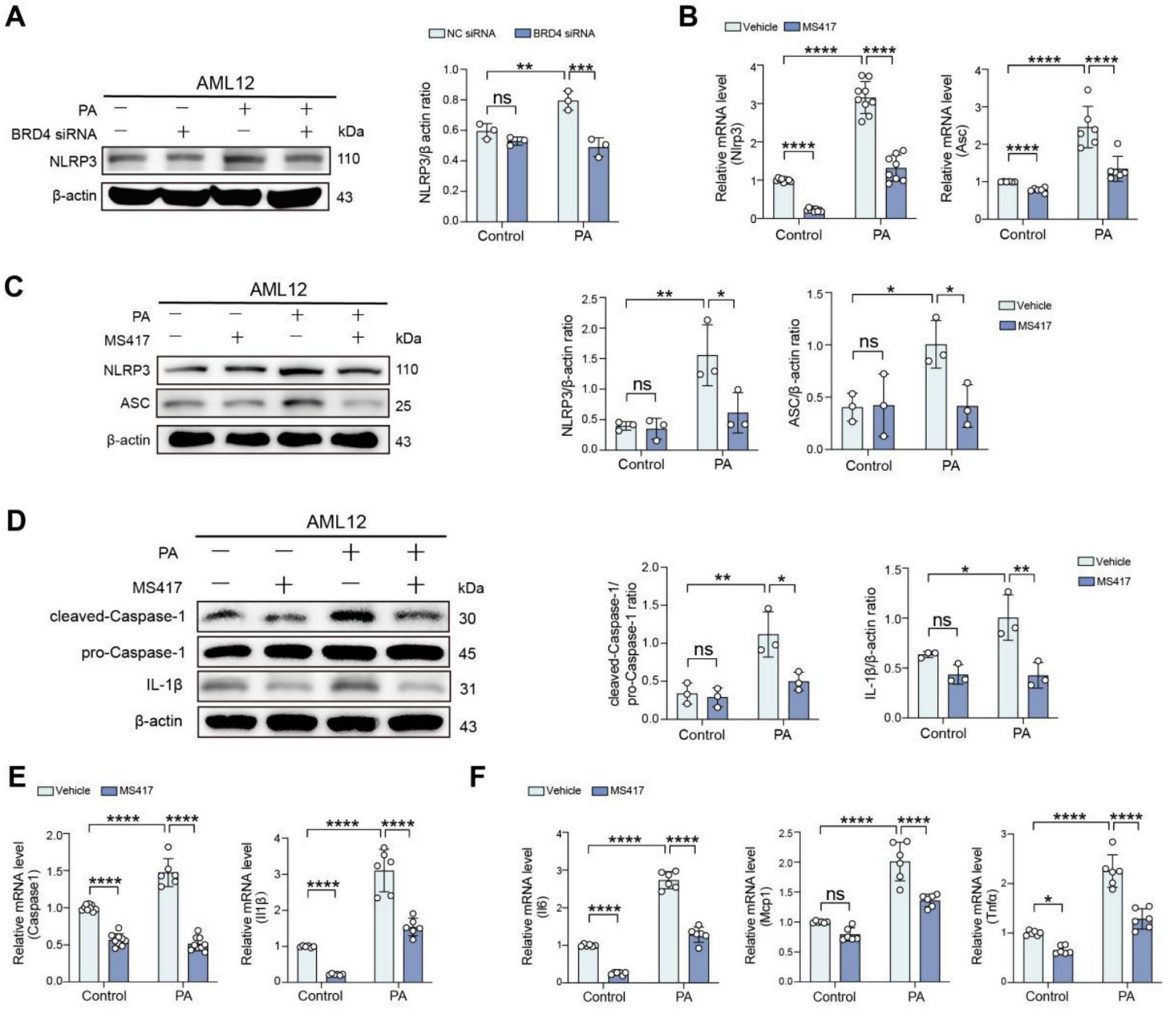
Targeting BRD4 suppresses the NLRP3 inflammasome activation during hepatocellular lipotoxicity. **A** AML12 hepatocytes were transfected with NC siRNA or BRD4 siRNA. 48 h after transfection, cells were exposed to PA (500µM) or BSA (control) for 24 h. Western blot for NLRP3 expression in cells (right) and densitometric analysis (left). β-actin was used as loading control and results as relative to β-actin (*n* = 3). **B-F** AML12 hepatocytes were exposed to PA or BSA for 24 h in the presence MS417 (50 nM) or vehicle. **B** Quantitative RT-PCR was performed to determine the mRNA expression of *Nlrp3* and *Asc* in AML12 cells from indicated treatment. Results were normalized to β-actin mRNA and expressed as fold change compared to control group. **C** Western blot for NLRP3 and ASC in cells from indicated treatment (left) and its densitometric analysis (right). β-actin was used as loading control and results were normalized relative to expression of β-actin. **D** Western blot for cleaved-Caspase-1, pro-Caspase-1 and IL-1β (left) and its Densitometric analysis (right). β-actin was used as loading control and results were normalized relative to expression of β-actin. **E** Quantitative RT-PCR was performed to determine the mRNA expression of *Caspase1* and *Il1β* in cells. Results were normalized to β-actin mRNA and expressed as fold change compared to control group. **F** Quantitative RT-PCR was performed to determine the mRNA expression of *Il6*, *Mcp1*, and *Tnfα* in hepatocytes. Results were normalized to β-actin mRNA and expressed as fold change compared to control group. Statistical significance is denoted by *p < 0.05, **p < 0.01, ***p < 0.001; ****p < 0.0001; ns indicates not significant

To explore how BRD4 contributes to hepatocellular lipotoxicity, we assessed the activation of NLRP3 inflammasome in hepatocytes upon PA stimulation. Given the NLRP3 binds to adaptor protein ASC, which in turn binds to pro-Caspase-1 to form and activate the NLRP3 inflammasome [14, 15]. Thus, we analyzed the expression of NLRP3 and ASC in hepatocytes during hepatocellular lipotoxicity in our in vitro model. We found that PA overload upon AML12 hepatocytes led to an increase in the level of NLRP3 expression as shown by Western blot. However, BRD4-deficient AML12 hepatocytes suppressed the increased level of NLRP3 expression in PA-induced hepatocytes (Fig. 2A). Here, we used MS417 to abrogate the BRD4 signaling in PA-induced hepatocellular lipotoxicity, and found that the upregulated expression of NLRP3 and ASC mRNA and protein induced by PA was significantly abrogated by MS417 as compared with vehicle-treated hepatocytes (Fig. 2B, C).

Given NLRP3 inflammasome assembly occurs that results in Caspase-1 activation, which cleaves pro-Caspase-1 into cleaved-Caspase-1, and then cleaves pro-IL-1β and pro-IL18 into their active forms (IL-1β and IL-18) [10–13], we thus examined the effect of MS417 on Caspase-1 activation and IL-1β release. Our result exhibited that PA-stimulated AML12 hepatocytes were associated with increased levels of IL-1β protein and the activation of Caspase-1, which was represented by the ratio of cleaved-Caspase-1/pro-Caspase-1 protein expression. However, MS417 administration effectively depressed the activation of Caspase-1 and inhibited the upregulated of IL-1β (Fig. 2D). Consistent with these results, the mRNA expression of *Caspase1* and *Il1β* in hepatocytes induced by PA was markedly inhibited by the treatment of MS417 (Fig. 2E). As the NLRP3 inflammasome activation is a key trigger of liver inflammation [13–15], we therefore evaluated inflammatory cytokines such as *Il*6, tumor necrosis factor-α (*Tnfα*) and monocyte chemoattractant protein 1 (*Mcp1*) in AML12 hepatocytes upon toxicity by PA. As expected, PA-induced hepatocyte lipotoxicity led to increased levels of *Il6*, *Mcp1*, and *Tnfα* mRNA. However, MS417 treatment led to a marked reduction in the levels of these inflammatory markers during hepatocellular lipotoxicity (Fig. 2F).

Taken together, targeting BRD4 suppresses the NLRP3 inflammasome activation in AML12 hepatocytes during hepatocellular lipotoxicity.

### Inhibition of BRD4 mitigates the GSDMD-mediated pyroptosis during hepatocellular lipotoxicity

**Fig. 3:**
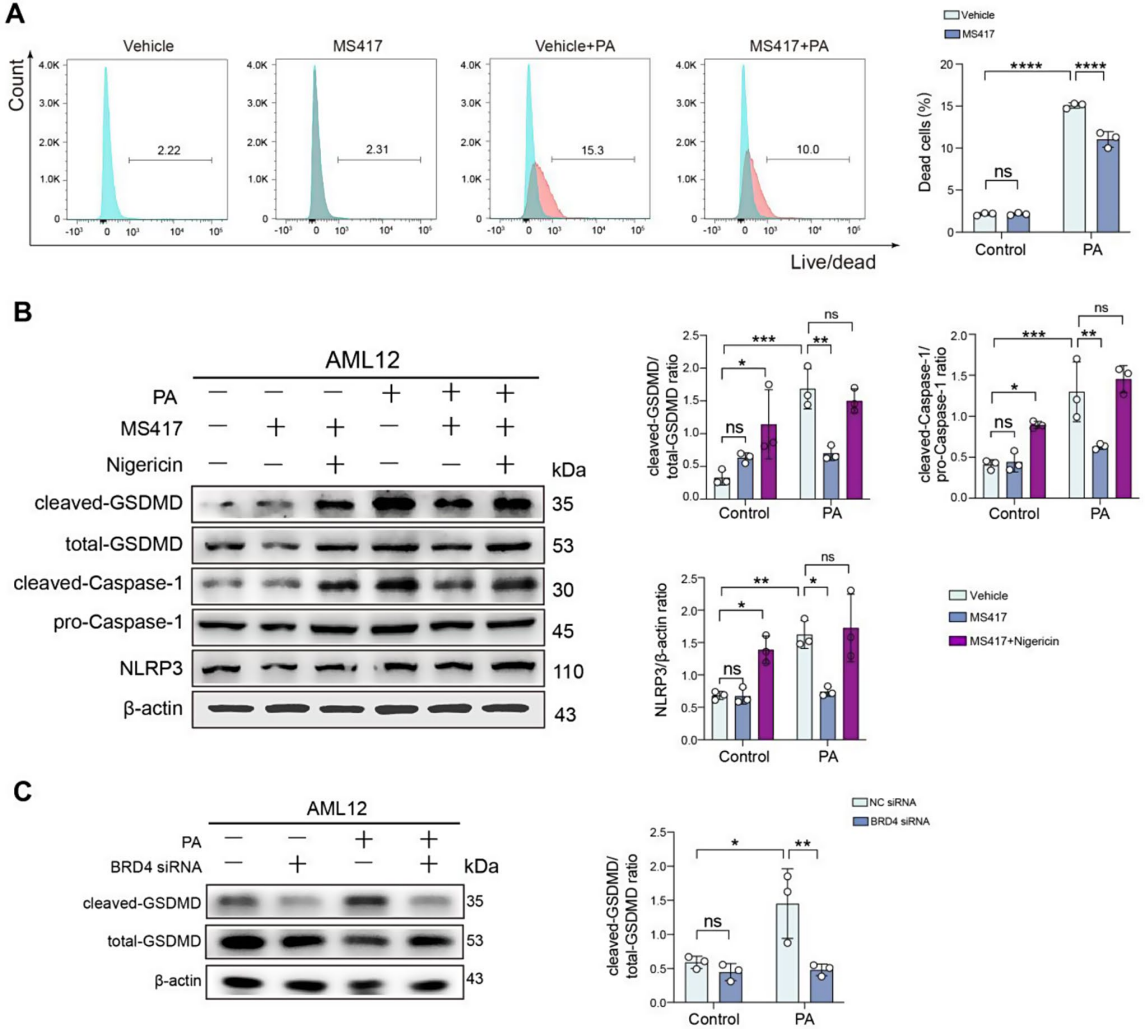
Inhibition of BRD4 mitigates the GSDMD-mediated pyroptosis during hepatocellular lipotoxicity. **A** AML12 hepatocytes were exposed to PA (500µM) or BSA (control) for 24 h in the presence of MS417 (50nM) or vehicle. Hepatocytes death was evaluated by flow cytometry using fixable Viability Dye 506 and analysed by FlowJo 10.8.1. **B** Western blot for cleaved-GSDMD, total-GSDMD, cleaved-Caspase-1, pro-Caspase-1, and NLRP3 in AML12 hepatocytes from indicated treatment groups and its densitometric analysis. Results were normalized relative to expression of β-actin (*n* = 3). **C** AML12 hepatocytes were transfected with NC siRNA or BRD4 siRNA. 48 h after transfection, cells were exposed to PA or vehicle for 24 h. Western blot for cleaved-GSDMD and total-GSDMD in cells lysates from indicated treatment (left) and its densitometric analysis (right). β-actin was used as loading control and results presented as the ratio cleaved-GSDMD to total-GSDMD (*n* = 3). Statistical significance is denoted by *p < 0.05, **p < 0.01, ***p < 0.001; ****p < 0.0001; ns indicates not significant

We investigated that the effect of BRD4 inhibitor MS417 on cell death in an in vitro model of hepatocellular lipotoxicity using AML12 hepatocytes; and cell death was evaluated by flow cytometry using fixable Viability Dye 506. Our results exhibited that the ratio of dead cells to live cells was significantly increased under PA toxicity to hepatocytes; however, this increased ratio was significantly reduced by MS417 treatment (Fig. 3A), indicating BRD4 playing a major contributor to the death of hepatocytes during lipotoxicity.

Recent reports demonstrated that the NLRP3 inflammasome activation led to cellular pyroptosis, an executor of pyroptotic cell death, through membrane rupture and the release of proinflammatory intracellular contents mediated by cleavage of GSDMD [11, 13]. We next asked whether BRD4 is involved in modulating pyropotic cell death during lipotoxicity. Thus, we assessed the expression of total and cleaved GSDMD in PA-treated AML12 hepatocytes. Our result showed that the expression of cleaved GSDMD was notably enhanced in PA-stimulated AML12 hepatocytes. However, the enhanced trend was restrained by treatment with MS417 (Fig. 3B), indicating that MS417 inhibited the pyroptosis of hepatocytes during hepatocellular lipotoxicity. Additionally, our Western blot analysis exhibited that MS417 treatment remarkably blocked the NLRP3 inflammasome activation by nigericin, which served as a positive control (Fig. 3B). It has been established that the Caspase-1-mediated classical pyroptosis plays an important role in the development and progression of MASLD and MASH [9–11]. We then asked whether BRD4 could inhibit this classical pyroptosis during hepatocellular lipotoxicity. As expected, our Western blotting exhibited that the protein level of cleaved-Caspase-1 was remarkably enhanced in PA-induced AML12 hepatocytes compared with that BSA-induced the same cells. However, the enhanced expression levels of these pyroptosis-related proteins were restrained by the treatment of MS417 (Fig. 3B). In line with this result, our results also demonstrated that siRNA-mediated BRD4 knockdown in AML12 hepatocytes under PA toxicity reduced the expression of the cleaved-GSDMD (Fig. 3C).

Taken together, MS417 treatment attenuated the classical Caspase-1-mediated pyroptosis in PA-induced AML12 hepatocellular lipotoxicity.

### BRD4 triggers VDAC1 oligomerization during hepatocellular lipotoxicity

**Fig. 4:**
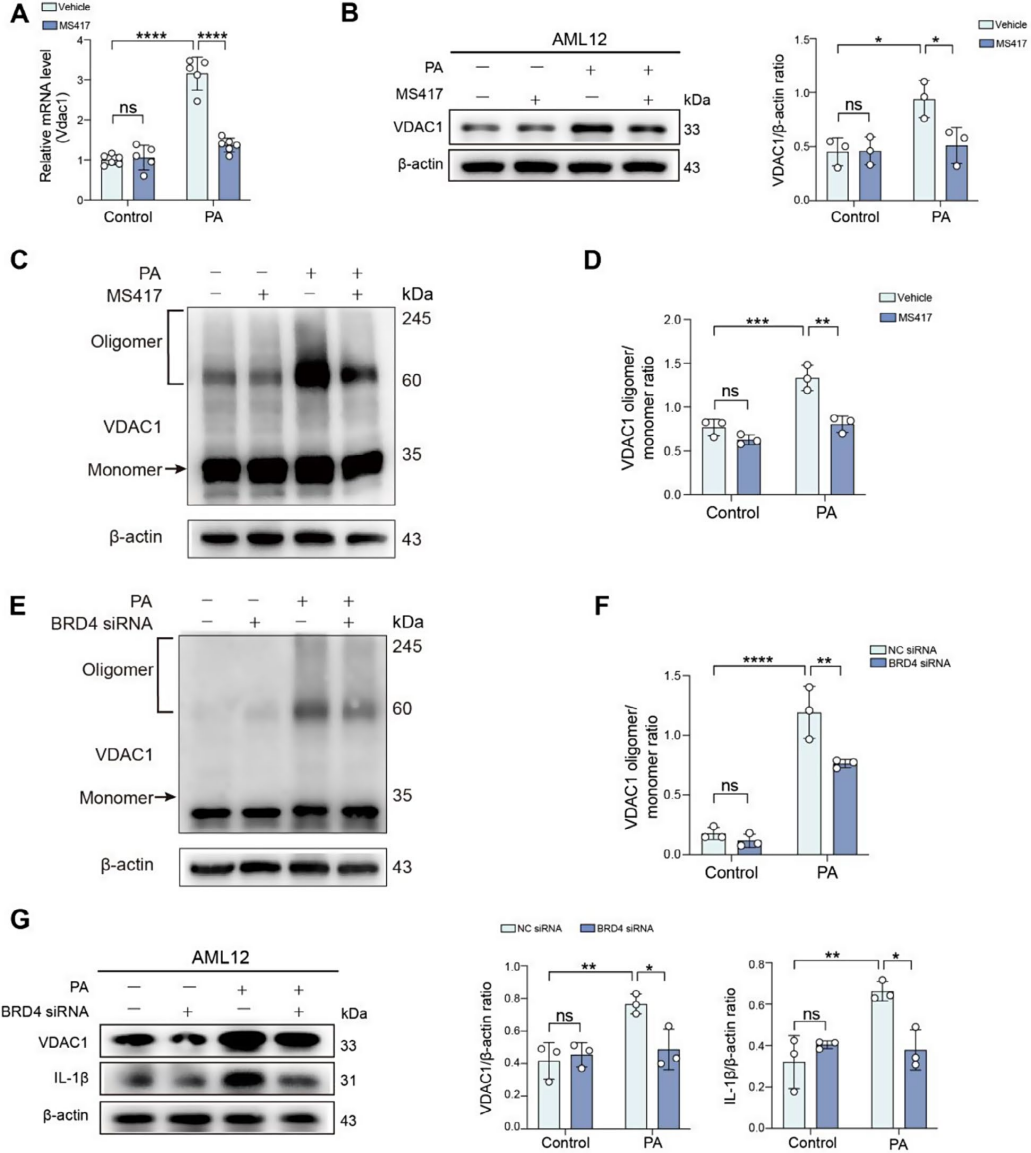
BRD4 triggers VDAC1 oligomerization during hepatocellular lipotoxicity. **A-D** AML12 hepatocytes were exposed to PA (500µM) or BSA (control) for 24 h in the presence MS417 (50 nM) or vehicle. **A** Quantitative RT-PCR was performed to determine the mRNA expression of *Vdac1* in cells. Results were normalized to β-actin mRNA and expressed as fold change compared to control group. **B** Western blot for VDAC1 in cell lysates (left) and its densitometric analysis (right); β-actin was used as loading control and results as relative to β-actin (*n* = 3). **C** Western blot for the VDAC1 oligomerization in AML12 hepatocytes from indicated groups with the treatment of EGS (600µM, 30 min) before extracting proteins. **D** The ratio of VDAC1 oligomer/monomer in (C) analysed by ImageJ. **E** Western blot for the VDAC1 oligomerization in AML12 hepatocytes. The cells were transfected with NC siRNA or BRD4 siRNA; 48 h after transfection, the cells were challenged with PA or BSA (control) for another 24 h and were treated with EGS (600µM, 30 min) before extracting proteins. **F** The ratio of VDAC1 oligomer/monomer in **(E)** analysed by ImageJ. **G** AML12 hepatocytes were transfected with NC siRNA NC or BRD4 siRNA. 48 h after transfection, cells were exposed to PA or BSA (control) for 24 h. Western blot for VDAC1 and IL-1β in cell lysates from indicated treatment group (left) and its densitometric analysis (right). β-actin was used as loading control and results as relative to β-actin. Statistical significance is denoted by *p < 0.05, **p < 0.01, ***p < 0.001; ****p < 0.0001; ns indicates not significant

As mitochondrial protein VDAC oligomerization is required for the NLRP3 inflammasome activation [14, 15], we asked whether BRD4 can trigger VDAC1 oligomerization during hepatocellular lipotoxicity. After AML12 hepatocytes challenged with PA for 24 h, we found that the expression of VDAC1 gene and protein was upregulated; but MS417 treatment abrogated this enhanced expression (Fig. 4A, B). Next, we measured the oligomerization of VDAC1 using the VDAC1 cross-linking assay. Our result exhibited that PA-induced hepatocyte lipotoxicity led to increasing the oligomerization of VDAC1, which was significantly inhibited by MS417 administration (Fig. 4C, D). Similarly, our results also demonstrated that BRD4 deficiency AML12 hepatocytes caused a reduction of VDAC1 protein level and blocked the oligomerization of VDAC1 during the cells exposed to toxicity by PA (Fig. 4E-G). Additionally, as a consequence of BRD4 knockdown in AML12 hepatocytes, the expression of IL-1β protein was highly diminished in response to PA overload in PA-induced hepatocyte liptoxicity (Fig. 4G). Together, our results robustly indicate that BRD4 plays a key role in upregulating VDAC1 expression and subsequently triggering its oligomerization during hepatocellular lipotoxicity.

### BRD4 upregulates VDAC and GSDMD expression via H3K27ac in the promoter regions

**Fig. 5:**
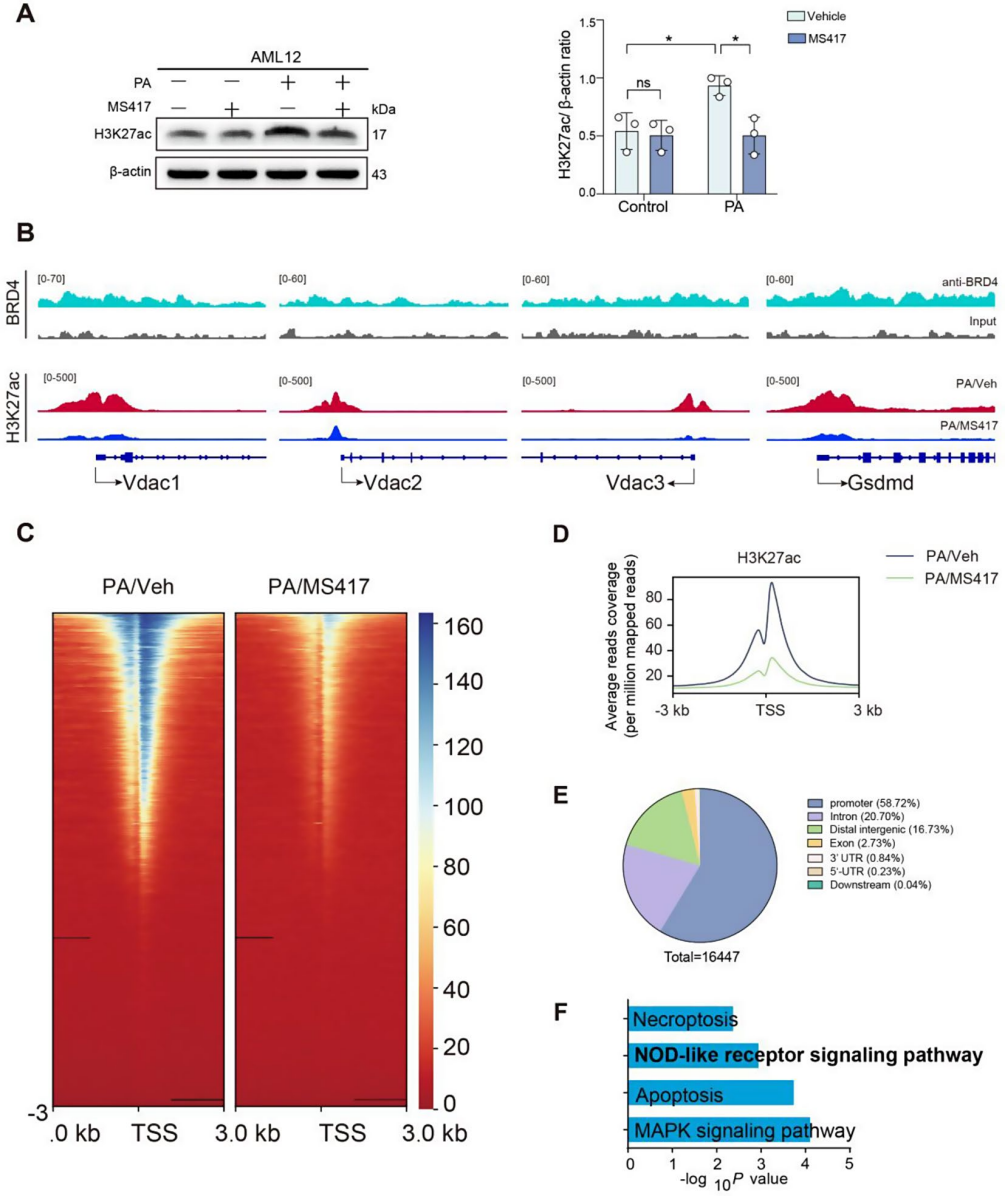
BRD4 upregulates VDAC and GSDMD expression via H3K27ac in the promoter region during hepatocellular lipotoxicity. AML12 hepatocytes were exposed to PA (500µM) or BSA (control) for 24 h in the presence of MS417 (50nM) or vehicle (Veh). **A** Western blot for H3K27ac in PA-induced AML12 cells indicated treatment (left) and densitometric analysis (right). β-actin was used as loading and results as relative to β-actin. Representation of *n* = 3 independent experiments. **B** Normalized ChIP-seq tracks showing BRD4 and H3K27ac protein binding for Vdac1, Vdac2, Vdac3, and Gsdmd in AML12 hepatocytes under indicated treatment using IGV 2.16.2. Upper line: the comparative ChIP-seq assay of BRD4 between binding anti-BRD4 group (blue) and BSA (Input) group (grey) based on AML12 induced by PA; lower line: the comparative ChIP-seq Assay of H3K27ac between PA/Veh group (red) and PA/MS417 group (blue). **C** Heatmap showing the signal intensity of H3K27ac (3.0 kb) around the transcription start site (TSS) of each differentially activated enhancer ordered by mean signal. **D** Average reads coverage within 3.0 kb from the TSS of H3K27ac in hepatocytes indicated treatment. PA/Veh (black line) and PA/MAS417 (green line). **E** Pie chart of genomic annotation depicting the genome-wide distribution of the differentially binding region is shown. **F** Ingenuity KEGG pathway analysis depicting the top pathways regulated by differential genes obtained from H3K27ac ChIP-seq analysis. Statistical significance is denoted by *p < 0.05, ns indicates not significant

BRD4 has been established to recognize the acetylation of histone H3 at lysine 27 (H3K27ac) through the acetyl-lysine binding domain of genes and regulate target genes expression [33, 34]. Thus, we measured the level of H3K27ac in PA-treated AML12 hepatocytes using Western blot. As expected, we found the level of active epigenetic mark H3K27ac was increased in PA-induced AML12 hepatocytes; while this increased level of H3K27ac was blocked by MS417 treatment as compared with vehicle treatment (Fig. 5A). Furthermore, we found that all three subtypes of *Vdac* (*Vdac1*, *Vdac2* and *Vdac3*) were bound by H3K27ac at the regions of promoter of AML12 hepatocytes. Under the treatment of MS417, the intensity of H3K27ac signals bound with *Vdac1*, *Vdac2*, and *Vdac3* were all significantly attenuated, with *Vdac1* being the most statistically different (Fig. 5B).

As VDAC1 expression was regulated by BRD4 in PA-treated AML12 hepatocytes (Fig. 4A), we speculate that VDAC1 might be a molecular target of BRD4. We firstly determined the genome-wide DNA binding regions of BRD4 in AML12 hepatocytes upon PA toxicity using ChIP-seq assay. Visualizing the BRD4 ChIP-seq peak shape by IGV (Integrative Genomics Viewer) and seeking out the target genes, we found that BRD4 highly occupied the promoter regions of the three subtypes of *Vdac* (*Vdca1*, *Vdac2*, and *Vdac3*), suggesting that BRD4 protein could locate in the promoter regions of *Vdac* to a certain extent, which prerequisites for BRD4 to fulfill its epigenetic regulatory function on VDAC in PA-treated AML12 hepatocytes (Fig. 5B).

Next, to further explore the epigenetic regulatory mechanisms of BRD4, we performed comparative H3K27ac ChIP-seq assay in PA-induced AML12 hepatocytes between upon vehicle-treatment (PA/Veh) and MS417-treatment (PA/MS417). For all the resulting peaks, an average normalized reads coverage was computed and visualized to show that the enrichment intensity of H3K27ac was significantly lower in the MS417-treated hepatocytes than that in the vehicle-treated hepatocytes (Fig. 5C, D). In accordance with our finding of lower total H3K27ac levels, we identified 1,6374 regulatory elements were lost (decreased H3K27ac) and 73 were gained (increased H3K27ac) in the MS417-treated to PA-induced AML12 hepatocytes as compared with that in vehicle-treated to PA-induced AML12 hepatocytes (Fig. 5E). Moreover, the annotation of these regulatory elements revealed that 58.72% of the differentially enriched H3K27ac ChIP-seq peaks were located in the regions of promoter (Fig. 5E).

Altogether, our data indicate that BRD4 promoted VDAC transcription by regulating the binding of H3K27ac to the *Vdac* promoters, thereby triggering VDAC expression and subsequent oligomerization, which in turn exerts a key role in activating the NLRP3 inflammasome and promoting hepatocyte pyroptosis. Notably, KEGG pathway analysis of the differentially bound genes revealed NOD-like receptor signaling pathway as one of the major influenced network (Fig. 5F), which underpinned our results, that BRD4 epigenetically regulates the NLRP pathway via H3K27ac.

Intriguingly, we also noted that *Gsdmd* was found to bind to both BRD4 and H3K27ac at the promoter region resulting in increased expression of GSDMD; and the intensity of Gsdmd-bound H3K27ac signals was remarkably weakened in the PA/MS417-treated group compared to the PA/Vehicle-treated group (Fig. 5B). GSDMD acts as an executor protein of pyroptosis that is initiated after the NLRP3 inflammasome activation.

Taken together, our data demonstrated that BRD4 promotes GSDMD processing not only through the VDAC/NLRP3 signaling pathway but also by directly enhancing the transcription of GSDMD.

### BRD4 is up-regulated in the liver samples from patients with MASH

**Fig. 6:**
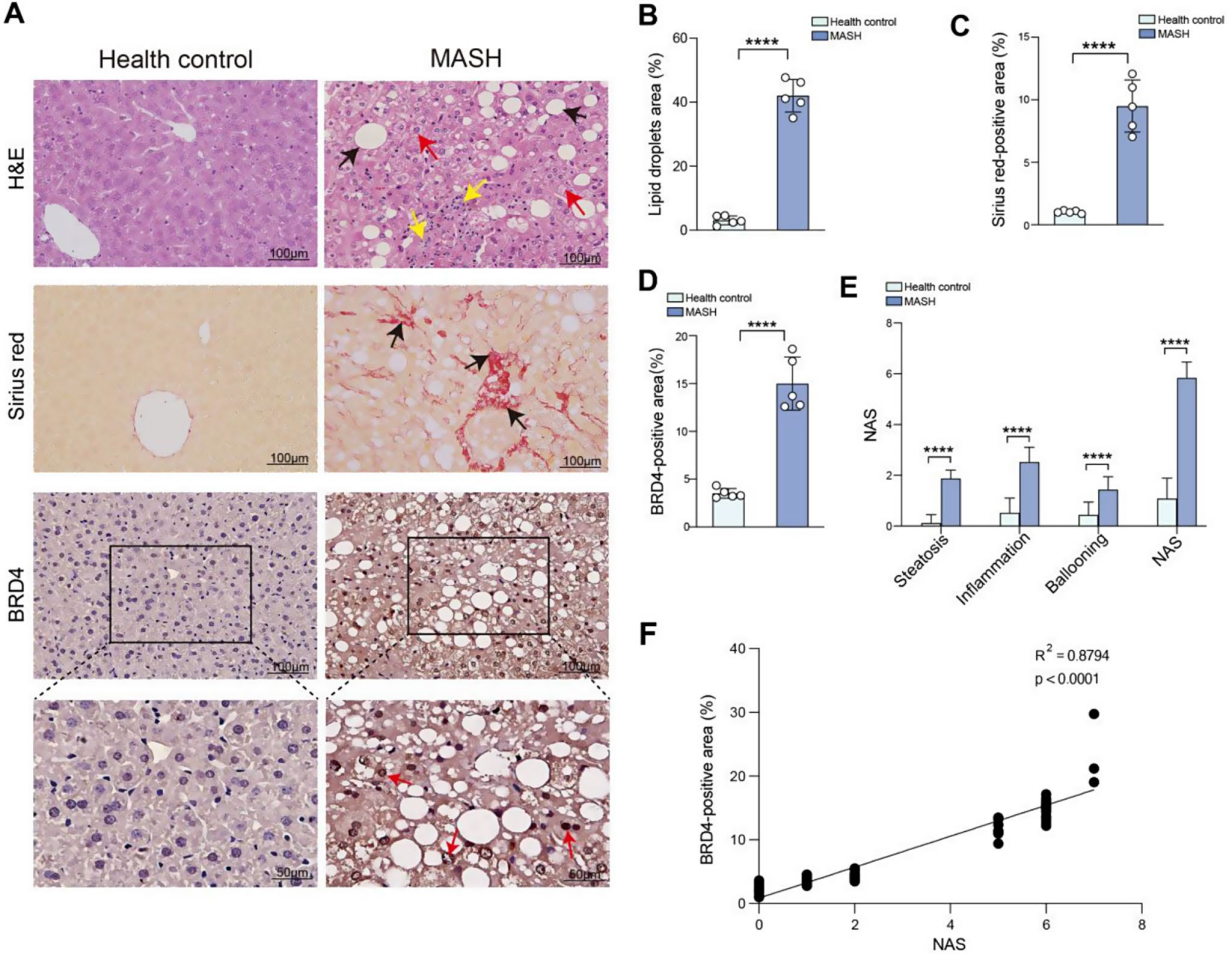
BRD4 is up-regulated in the liver samples from patients with MASH. **A** Representative images of liver sections stained with H&E, Sirius red, and anti-BRD4 antibody (Scale bars: 100 μm; original magnification: ×200). Boxes indicate regions of higher magnification (Scale bars: 50 μm; original magnification: ×400). **B** Quantitative analysis of lipid droplet areas in liver sections based on H&E staining (*n* = 5). **C** Fibrotic areas in liver sections based on Sirius Red staining (*n* = 5). **D** Areas of BRD4-positive cells (%) per field (*n* = 5) were analyzed by ImageJ. **E** NAFLD activity score (NAS) of normal controls (*n* = 25) or MASH patients (*n* = 25). **F** Relation between BRD4-positive area and NAS score. Statistical significance is denoted by ****p < 0.0001

To determine whether BRD4 is involved in the pathology of human MASLD/MASH, liver sections from clinical samples of MASLD patients were stained with BRD4 antibody. MASLD patients were diagnosed blindly by two pathologists into the following categories: healthy control and MASH with fibrosis. H&E and Sirius red staining in liver sections were conducted to indicate the extent of hepatic steatosis, intralobular inflammation, hepatocyte ballooning, and fibrosis (Fig. 6A-C). As expected, the scores of liver steatosis, intralobular inflammation, and hepatocellular ballooning in MASH patients were considerably higher than those of healthy controls based on the NAS scores (Fig. 6E). As shown in Sirius red staining of liver sections, collagen deposition was substantially enhanced in the MASH patients’ liver tissues compared to controls (Fig. 6A, C). IHC analysis indicated that BRD4 expression was weak in healthy livers and remarkably increased in hepatocytes from MASH fibrosis patients. We also noted that the BRD4 largely localized in hepatocytes of MASH livers (Fig. 6A). Consistent with this observation, the area of BRD4^+^ cells in liver sections from MASH patients was highly increased than that in healthy control (Fig. 6D). Further, the expression levels of BRD4 in the liver increased along with the progression of MASH liver pathology, according to the Pearson correlation analysis of BRD4 positivity in liver tissue and NAS score in each patient, revealing a positive linear correlation between BRD4 positivity and NAS score (R2 = 0.8972, *p* < 0.0001) (Fig. 6F). Taken together, these data in humans strongly indicated that BRD4 might be involved in pathogenesis of MASH and fibrosis.

### Hepatic BRD4, NLRP3, GSDMD, and VDAC1 expression enhanced in a diet-induced MASH mice

**Fig. 7:**
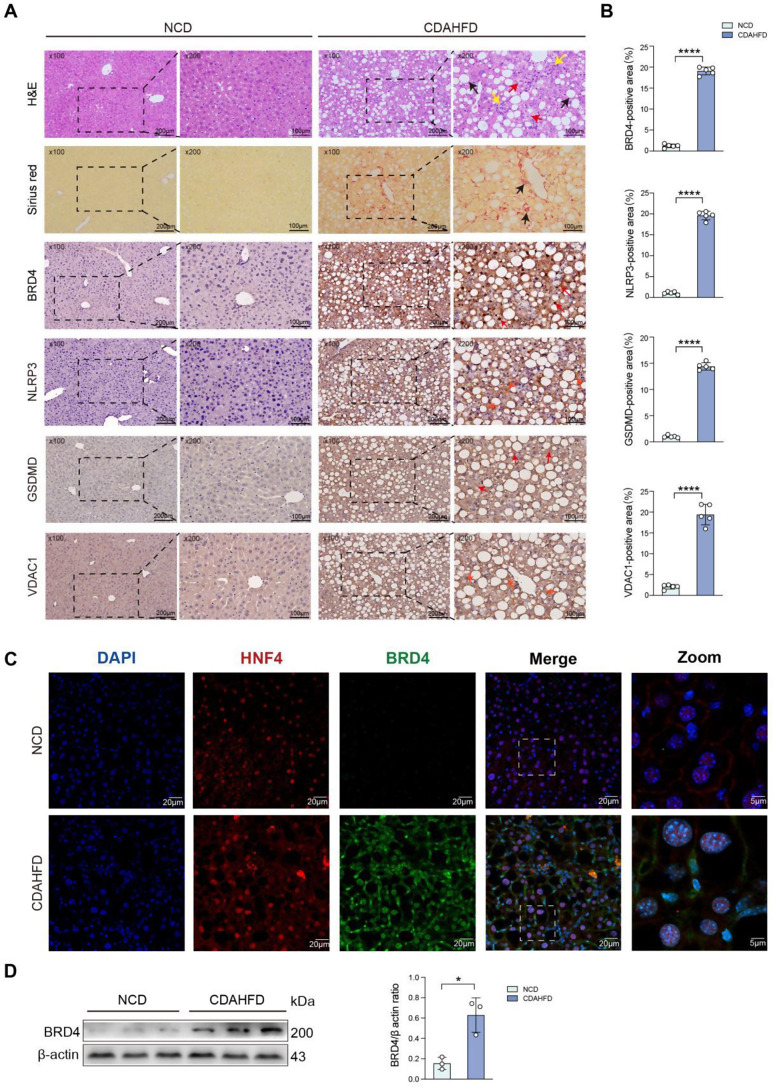
Hepatic BRD4, NLRP3, GSDMD, and VDAC1 expression enhanced in a diet-induced MASH mice. **A** Representative images of liver sections stained with H&E, Sirius red, and IHC stained for BRD4, NLRP3, GSDMD, and VDAC1. Insert showing the typical lobular inflammation (yellow arrow), pericellular fibrosis (black arrow), and positively stained cells (red arrow). Scale bars: 200 μm in left row, 100 μm in right row. **B** Percentage of the positive areas for BRD4, NLRP3, GSDMD, and VDAC1 in liver sections from indicated mice (*n* = 5 mice/group). **C** Representative immunofluorescence images and detection of BRD4 (green), HNF4 (red) in liver sections; nuclei stained with DAPI (blue). Scale bar = 20 μm. Boxes indicate regions of higher magnification. **D** Left: Western blot analysis of BRD4 levels in livers from normal chow diet (NCD) or CDAHFD-fed mice; β-actin was used as loading control. Right: Densitometric analysis, results as relative to β-actin. Statistical significance is denoted by *p < 0.05; ****p < 0.0001

To confirm the finding in clinical liver sections, we further used IHC to assess the expression of BRD4 in the livers of mice fed with CDAHFD. As shown in H&E and Sirius red staining of liver sections, feeding C57BL/6J mice with CDAHFD for 8 weeks induced hepatic steatosis, inflammation, and fibrosis (Fig. 7A). Meanwhile, the livers from mice fed with NCD exhibited no signs of hepatic steatosis, inflammatory response, or fibrosis (Fig. 7A). IHC exhibited that the hepatic BRD4 remarkably enhanced in MASH mice as compared with NCD-fed mice. Notably, BRD4 protein was mostly localized in hepatocytes, and the BRD4 positive region expanded from the nucleus to a considerable cytoplasmic region, corroborating the early clinical results (Fig. 7A, B). Supporting this, our double labeling immunofluorescence microscopy exhibited that the BRD4 immunoreactivity in MASH liver was mostly co-localized with hepatic nuclear factor 4 (HNF4), which is a specific marker of hepatocyte; this result revealed that BRD4 was expressed predominantly in hepatocytes (Fig. 7C). BRD4 protein expression was substantially higher in the liver from CDAHFD-fed mice compared with NCD-fed control mice (Fig. 7D). We also assessed the expression of NLRP3, VDAC1, and GSDMD in liver sections from mice by IHC. Our result exhibited stronger staining of NLRP3, VDAC1, and GSDMD in liver sections from MASH mice than that in NCD-fed control mice (Fig. 7A). Accordingly, the areas of NLRP-positive, VDAC1-positive, and GSDMD-positive cells in MASH liver sections were higher than that in normal liver sections, respectively (Fig. 7B). Altogether, our finding demonstrated that the expression of BRD4, NLRP3, GSDMD, and VDAC1 were increased in the liver from MASH mice.

## Discussion

MASH is the severer stage of MASLD that can progress to irreversible fibrosis, cirrhosis, and eventually HCC and has emerged as the leading cause of liver failure [1, 35]. Despite MASH being a rising global health threat with clear links to obesity, there are still limited agents for its treatment [3, 35]. Therefore, identifying novel therapeutic targets is urgently needed. In this study, we demonstrate that BRD4 plays a major contribution in the deposition of lipid droplets, NLRP3 inflammasome activation, and hepatocyte pyroptosis in the development of MASH through epigenetic regulation VDAC and GSDMD expression. Hence, our research offers a fresh epigenetic understanding of how BRD4 triggers the NLRP3/pyroptosis pathway and raises the possibility that the BRD4/VDAC1/NLRP3 signaling axis could be used as a potential target for MASH therapy.

Recent studies have demonstrated that MASLD is a complicated metabolic disorder, with both genetic and epigenetic factors influencing its development and progression [16, 36]. Aberrant epigenetic alterations can cause inappropriate gene expression and promote a broad range of chronic diseases including MASLD/MASH [2, 16, 36]. It has been reported that BRD4 senses hyper-acetylated chromatin sites and accumulates on transcriptionally active regulatory, thereby controlling gene transcription [37]. Here, our findings exhibited that the levels of BRD4 expression were enhanced in MASLD and MASH livers from patients and mice. Notably, in MASH livers, BRD4 is primarily found in hepatocytes (Figs. 6A and 7A and C). In line with these findings in liver sections, PA-treated hepatocytes led to a markedly upexpression of BRD4 (Fig. 1A, B). Therefore, our results indicate that BRD4 might contribute to lipid metabolism imbalance and MASLD progression.

Furthermore, using a cellular model of hepatic lipotoxicity, we found that targeting BRD4 by genetic knockdown or the selective BRD4 inhibitor effectively protected against hepatosteatosis (Fig. 1C-H). Consistently, inhibition of BRD4 with MS417 led to a significant reduction in levels of genes-related lipogenesis (*Acc1*, *Srebp1c*, and *Fasn*) in PA-induced AML12 hepatocytes lipotoxicity (Fig. 1I). Indeed, several previous reports have shown that BRD4 modulates hepatic lipid metabolism and participates in fructose-inducible fatty liver and high-fat diet-induced obesity [23, 38]. In our current study, we highlighted the events and consequences of lipid metabolic dysfunction posed by BRD4 in hepatocytes during MASH pathogenesis. Importantly, inhibition of BRD4 effectively blocked the activation of NLRP3 inflammasome and subsequently reduced hepatocyte pyroptosis, as well as limited inflammatory cytokines such as *Il*6, *Tnfα* and *Mcp1* in AML12 hepatocytes upon toxicity by PA (Figs. 2 and 3). Notably, accumulating evidence supported that the activation of aberrant NLRP3 inflammasome plays a pivotal role in MASH-related liver inflammation and fibrosis [6, 7, 10]. Once the activation of the NLRP3 inflammasome, pro-Caspase-1 would be activated and subsequently lead to IL-1β and IL-18 inflammatory cytokines maturation and secretion, therefore triggering the phenotype of pyroptosis [39]. Thus, our data suggests that BRD4 promotes the pathology of MASH through activating the NLRP3 inflammasome and promoting hepatocyte pyroptosis; and a better understanding molecular basis of how BRD4 activates the NLRP3 inflammasome in MASH is of importance for more effective clinical translation in the future.

It has been established that VDAC1 is essential for the NLRP3 inflammasome and its inhibition abrogates the NLRP3 inflammasome activation in a mitochondrial Ca^2+^- and ROS-dependent manner [14, 15]. Furthermore, VDAC1 oligomerization is identified as an upstream of NLRP3 inflammasome assembly [15, 30]. Here, we found inhibition of BRD4 with MS417 not only reduced the expression of VDAC1, but also inhibited the oligomerization of VDAC1 (Fig. 4). Moreover, our ChIP-seq data showed that *Vdac* (*Vdac1*, *Vdac2*, and *Vdac3*) transcription is associated with the acetylation of histone H3K27ac regulated by BRD4 (Fig. 5). Accompanying enhanced BRD4 expression, the levels of H3K27ac were increased in PA-induced AML12 hepatocytes. Remarkably, blocking BRD4 by MS417 led to a significant reduction in the level of H3K27ac, which downregulated the expression of VDAC1 and subsequently inhibited VDAC1 oligomerization (Fig. 4), therefore suppressing the activation of the NLRP3 inflammasome and the GASDMD-mediated pyroptosis. Once VDAC1 were oligomerized, oxidized-mitochondrial DNA would efflux from mitochondria via VDAC1 oligomers and binds to cytosolic NLRP3, activating NLRP3 inflammasome [30]. Conversely, inhibition of VDAC oligomerization led to impairing the association between VDAC and NLRP3 which subsequently abrogated the activation of the NLRP3 inflammasome [15]. Notably, several studies revealed that the increase in expression of VDAC1 led to the oligomerization of VDAC and also mediated apoptosis [40–42].

In this study, we also found that higher level of histone mark H3K27ac located in the promoter regions of *Gsdmd* and enhanced expression of GSDMD in PA-treated AML12 hepatocytes (Fig. 5B); and this may directly induce GSDMD-mediated hepatocyte pyroptosis during lipotoxicity. However, MS417 inhibited the expression of GSDMD by limiting the level of histone mark H3K27ac at the promoter regions (Fig. 5A, B). In addition, GSDMD is upregulated in liver sections from MASH mice (Fig. 7A, B). BRD4 inhibitors are known to suppress gene expression by dissociating BRD4 from active chromatin marks, especially histone H3K27ac [33, 34, 43]. Our data supported the concept that H3K27ac functions as the marker of active enhancers or super-enhancers that participate in the transcriptional activation of multiple genes [44, 45].

Several limitations of the present study should be acknowledged. First, primary and immortalized mouse hepatocytes were used to explore the role of BRD4 in the pathogenesis of MASH, and this in vitro cellular model may not be representative of in vivo metabolic conditions. To this end, further studies using mouse models are needed. Second, we still have little knowledge of the effect of BRD4 on human lipid metabolism. Our in vitro study only used mouse hepatocytes, and supplementation with human hepatocytes might have made the study more convincing. Finally, besides VDAC1, several other upstream regulators have been reported to be involved in the activation of the NLRP3 inflammasome, such as IRGB10, IRE1α, IRF8, and mtDNA fragments [15, 46], but we did not assess these molecules in this study. It would be interesting to investigate whether these molecules control NLRP3 inflammasome activation via BRD4 during MASH development.

In conclusion, our findings demonstrate that BRD4 is critical for PA-induced hepatotoxicity by stimulating the NLRP3 inflammasome and facilitating GSDMD-mediated pyroptosis, which was, at least partially, linked to the epigenetic controls of GSDMD and VDAC. Thus, our research offers fresh insights into the mechanics of the NLRP3 inflammasome activation and pyroptosis in hepatic lipotoxicity. It also raises the possibility that the BRD4 could be a target for MASLD/MASH management.

### Electronic supplementary material

Below is the link to the electronic supplementary material.

Supplementary Material 1

## Data Availability

All the data supporting the findings of this study are available within the manuscript. Data will be made available on reasonable request, not applicable for material.

## Abbreviations

ASC: Apoptosis-associated speck-like protein
BET: Bromodomain and extraterminal domain
BRD4: Bromodomain-containing protein 4
BSA: Bovine serum albumin
CDAHFD: Choline-deficient, L-amino acid-defined, high-fat diet
ChIP: Chromatin immunoprecipitation
ChIP-seq: ChIP sequence
GSDMD: Gasdermin D
HCC: Hepatocellular carcinoma
IHC: Immunohistochemical
IL-1β: Interleukin-1β
MASH: Metabolic dysfunction-associated steatohepatitis
MASLD: Metabolic dysfunction-associated steatotic liver disease
Mcp1: Monocyte chemoattractant protein 1
NAFLD: Nonalcoholic fatty liver disease
NAS: NAFLD activity score
NCD: Normal chow diet
NLRP3: Nod-like receptor family pyrin-containing protein 3
ORO: Oil Red O
PA: Palmitic acid
siRNA: Small interfering RNA
Tnfα: Tumor necrosis factor-α
VDAC: Voltage-dependent anion channel
