# Supplementary material for: Targeting BRD4 mitigates hepatocellular lipotoxicity by suppressing the NLRP3 inflammasome activation and GSDMD-mediated hepatocyte pyroptosis
Source: Cell Mol Life Sci. 2024 Jul 9;81(1):295. doi: 10.1007/s00018-024-05328-7 (PMC11335218; doi:10.1007/s00018-024-05328-7)
Supplement: Supplementary file 1 — Supplementary Material 1 [file 18_2024_5328_MOESM1_ESM.docx]

**Targeting BRD4 mitigates hepatocellular lipotoxicity** **by suppressing the NLRP3 inflammasome activation and GSDMD-mediated hepatocyte pyroptosis**

Fangyuan Chen^1, †^, Shuyu Li^1, †^, Min Liu^2^, Cheng Qian^3^, Zhiyu Shang^2^, Xu Song^4^, Wei Jiang^1,5, *^, Chuantao Tu^2, *^

^1^Department of Gastroenterology and Hepatology, Zhongshan Hospital, Fudan University, Shanghai, 200032, China.

^2^Department of Gastroenterology, Shanghai Public Health Clinical Center, Fudan University, Shanghai, 201508, China.

^3^Institute of Neuroscience and State Key Laboratory of Neuroscience, CAS Center for Excellence in Brain Science and Intelligence Technology, Chinese Academy of Sciences, Shanghai, 200031, China.

^4^Department of Pathology, Shanghai Public Health Clinical Center, Fudan University, Shanghai, 201508, China.

^5^Department of Gastroenterology and Hepatology, Zhongshan Hospital (Xiamen), Fudan University, 361015, China.

^†^Fangyuan Chen and Shuyu Li contributed equally to this work.

**Correspondence**

^*^Correspondence author.

E-mail address: [jiang.wei@zs-hospital.sh.cn](mailto:jiang.wei@zs-hospital.sh.cn) (Wei Jiang); tuchuantao@shaphc.org (Chuantao Tu)

| Antibody | Catalog Number | Vendors |
| --- | --- | --- |
| anti-β actin  anti-BRD4 | #EM21002  #13440 | Huabio  Cell Signaling Technology |
| anti-VDAC1 | # ab15895 | Abcam |
| anti-NLRP3 (for Western blot)  anti-NLRP3 (for IHC) | #ab263899  #T55651 | Abcam  Abmart |
| anti-ASC  anti-total and cleaved N-terminal GSMDM | #TP73812  #P30823 | Abmart  Abmart |
| anti-HNF4  anti-total and cleaved caspase1 | #3113  #PA3241 | Cell Signaling Technology  Abmart |
| anti-IL-1β  anti-Histone H3 | # 26048-1-AP  #4499 | Proteintech  Cell Signaling Technology |
| anti-Acetyl-Histone H3 (Lys27) (H3K27ac) | #8173 | Cell Signaling Technology |

**Table S1** All the antibodies in this study

**Table S2** The oligo sequences for siRNA in this study

|  | Sequence (5’~3’) |
| --- | --- |
| BRD4 siRNA1 (2158) | GCCGUGUAGUACACAUAAUTT |
|  | AUUAUGUGUACUACACGGCTT |
| BRD4 siRNA2 (2051) | GCCUCCCACAUAUGAAUCATT |
|  | UGAUUCAUAUGUGGGAGGCTT |
| RD4 siRNA3 (1862) | GCAGAACAAACCAAAGAAATT |
|  | UUUCUUUGGUUUGUUCUGCTT |
| NTC siRNA | UUCUCCGAACGUGUCACGUTT |
|  | ACGUGACACGUUCGGAGAATT |

BRD4 siRNA, Small interfering RNA targeting BRD4; NC siRNA, Non-targeted scrambled control siRNA.

**Table S3** Primer sequences used for quantitative PCR in this study

| Gene | Forward Primer（5’-3’） | Reverse Primer（5’-3’） |
| --- | --- | --- |
| *βactin* | GGCTGTATTCCCCTCCATCG | CCAGTTGGTAACAATGCCATGT |
| *BRD4*  *Tnfα* | GTGAGAAGCTAGGCCGTGTAG  CCCTCACACTCAGATCATCTTCT | AGGCAGGACCTGTTTCAGAGT  GCTACGACGTGGGCTACAG |
| *Il6* | TTAAAAACCTGGATCGGAACCAA | GCATTAGCTTCAGATTTACGGGT |
| *Il1β* | GCAACTGTTCCTGAACTCAACT | ATCTTTTGGGGTCCGTCAACT |
| *Mcp1*  *Srebp1* | TTAAAAACCTGGATCGGAACCAA  TGACCCGGCTATTCCGTGA | GCATTAGCTTCAGATTTACGGGT  CTGGGCTGAGCAATACAGTTC |
| *Fasn*  *Acc1*  *Nlrp3*  *Asc*  *Caspase1*  *Vdac1* | GGAGGTGGTGATAGCCGGTAT  GATGAACCATCTCCGTTGGC  TCGCAGCAAAGATCCACACAG  CTTGTCAGGGGATGAACTCAAAA  ACAAGGCACGGGACCTATG  CCCACATACGCCGATCTTGG | TGGGTAATCCATAGAGCCCAG  GACCCAATTATGAATCGGGAGTG  ATTACCCGCCCGAGAAAGG  GCCATACGACTCCAGATAGTAGC  TCCCAGTCAGTCCTGGAAATG  CCCACATACGCCGATCTTGG |


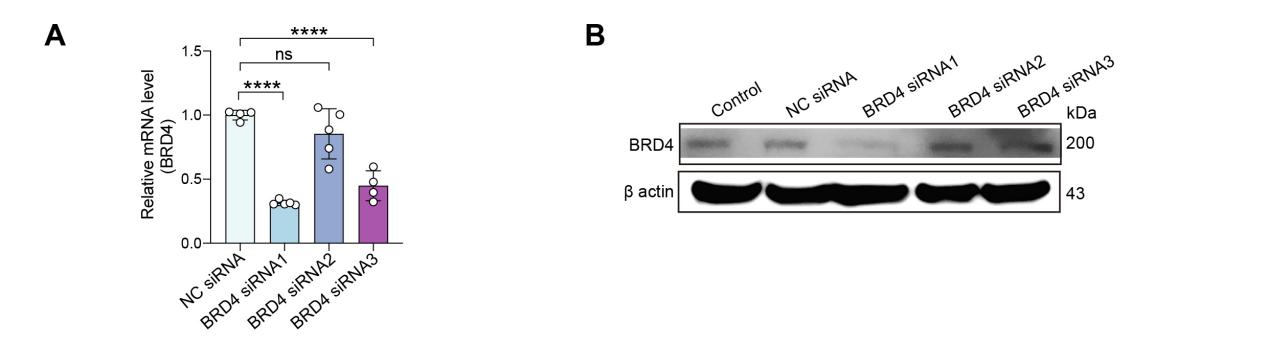


**Fig. S1** AML12 hepatocytes were transfected with NC siRNA or BRD4 siRNA #1, #2, and #3. 48 h after transfection, total RNA of cells was isolated for quantitative RT-PCR, and 72 h after transfection, cell proteins were lysed for western blot. **A** Quantitative RT-PCR was performed to determine the mRNA levels of BRD4 in the hepatocytes. Results were normalized to β-actin mRNA. **B** BRD4 protein in cell lysates was assessed by Western blot; β-actin was used as loading control.


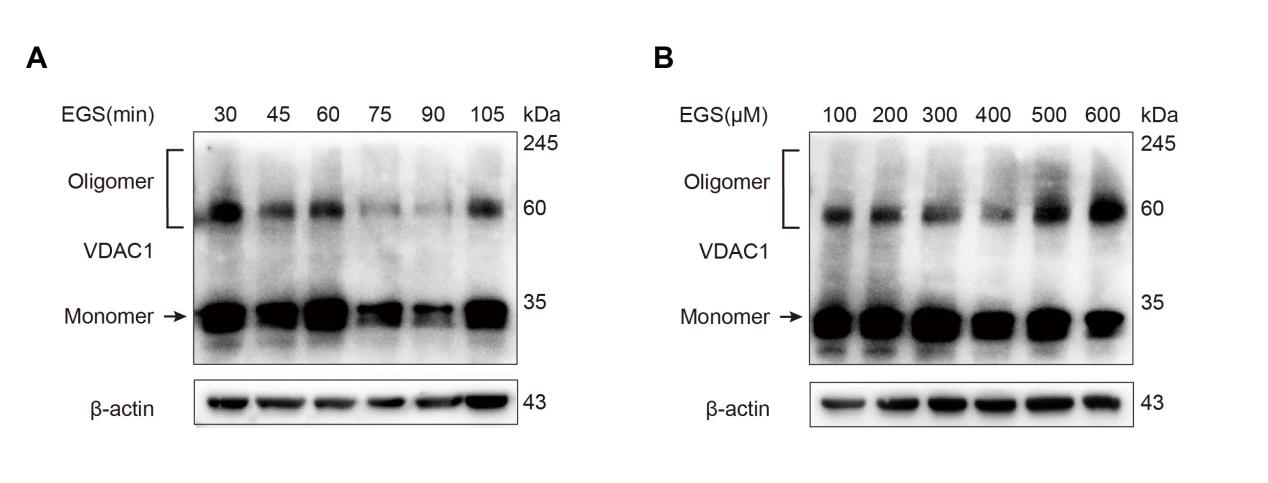


**Fig. S2** After being washed with PBS, AML12 was incubated with EGS for gradient time (A) and under gradient concentration (B). **A, B** VDAC1 protein monomer and oligomer in cell lysates was assessed by Western blot; β-actin was used as loading control.
